# Three-Dimensional Graphene Enhances Neural Stem Cell Proliferation Through Metabolic Regulation

**DOI:** 10.3389/fbioe.2019.00436

**Published:** 2020-01-08

**Authors:** Qiaojun Fang, Yuhua Zhang, Xiangbo Chen, He Li, Liya Cheng, Wenjuan Zhu, Zhong Zhang, Mingliang Tang, Wei Liu, Hui Wang, Tian Wang, Tie Shen, Renjie Chai

**Affiliations:** ^1^MOE Key Laboratory for Developmental Genes and Human Disease, Jiangsu Province High-Tech Key Laboratory for Bio-Medical Research, Institute of Life Sciences, Southeast University, Nanjing, China; ^2^Key Laboratory of Molecular Epigenetics of the Ministry of Education, Northeast Normal University, Changchun, China; ^3^Hangzhou Rongze Biotechnology Co., Ltd. Hangzhou, China; ^4^Department of Otolaryngology-Head and Neck Surgery, First Affiliated Hospital of Wenzhou Medical University, Wenzhou, China; ^5^Institute of Life Sciences, Anhui Agricultural University, Hefei, China; ^6^Zhangjiagang City First People's Hospital, The Affiliated Zhangjiagang Hospital of Suzhou University, Zhangjiagang, China; ^7^Department of Otolaryngology-Head and Neck Surgery, The Second Xiangya Hospital, Central South University, Changsha, China; ^8^Department of Otolaryngology Head and Neck Surgery, Shanghai Jiao Tong University Affiliated Sixth People's Hospital, Shanghai, China; ^9^Key Laboratory of Information and Computing Science Guizhou Province, Guizhou Normal University, Guiyang, China; ^10^Co-Innovation Center of Neuroregeneration, Nantong University, Nantong, China; ^11^Institute for Stem Cell and Regeneration, Chinese Academy of Science, Beijing, China; ^12^Beijing Key Laboratory of Neural Regeneration and Repair, Capital Medical University, Beijing, China

**Keywords:** three dimensional graphene, neural stem cell (NSC), metabolic pathway, proliferation, metabolites

## Abstract

Graphene consists of two-dimensional sp2-bonded carbon sheets, a single or a few layers thick, which has attracted considerable interest in recent years due to its good conductivity and biocompatibility. Three-dimensional graphene foam (3DG) has been demonstrated to be a robust scaffold for culturing neural stem cells (NSCs) *in vitro* that not only supports NSCs growth, but also maintains cells in a more active proliferative state than 2D graphene films and ordinary glass. In addition, 3DG can enhance NSCs differentiation into astrocytes and especially neurons. However, the underlying mechanisms behind 3DG's effects are still poorly understood. Metabolism is the fundamental characteristic of life and provides substances for building and powering the cell. Metabolic activity is tightly tied with the proliferation, differentiation, and self-renewal of stem cells. This study focused on the metabolic reconfiguration of stem cells induced by culturing on 3DG. This study established the correlation between metabolic reconfiguration metabolomics with NSCs cell proliferation rate on different scaffold. Several metabolic processes have been uncovered in association with the proliferation change of NSCs. Especially, culturing on 3DG triggered pathways that increased amino acid incorporation and enhanced glucose metabolism. These data suggested a potential association between graphene and pathways involved in Parkinson's disease. Our work provides a very useful starting point for further studies of NSC fate determination on 3DG.

## Introduction

Neural stem cells (NSCs) have received much attention in recent years as a therapeutic candidate for many neurodegenerative diseases such as Parkinson's disease (PD) and Alzheimer's disease (AD) (Soldner et al., 2009; Swistowski et al., 2010; Kang et al., 2016), and much research has focused on uncovering the detailed mechanisms behind NSCs fate determination. NSCs have two main properties, first, they have unlimited self-renewal capacity throughout the lifespan of the organism (Akesson et al., 2008; Campbell et al., 2014), and secondly they are multipotent and can differentiate into all types of cells within the neuro-ectodermal lineages of central nervous system, for example, glia cells and a variety of neurons (Chiasson et al., 1999). NSC fate is affected by extracellular and intracellular factors, especially the particular microenvironment in which the NSC is located as well as metabolic state of the cell (Kim et al., 2014). Several studies have reported that metabolic pathways are regulators of NSC proliferation and differentiation fate decisions, but the detailed mechanisms behind this regulation are still not fully understood.

Graphene has been studied as an useful nanomaterial in biomedical applications, stem cell research, cell imaging, drug delivery, and photo-thermal therapy due to it's excellent conductivity, stability, and biocompatibility (Geim, 2009; Kim et al., 2010; Zhu et al., 2010). The monolayer of carbon atoms in graphene can be arranged in two-dimensional lattices, fibers, and three-dimensional graphene (3DG) foams (Guo et al., 2016; Ma et al., 2016). Some publications have reported that graphene can enhance the neuronal differentiation of NSCs, and 3DG has been shown to provide suitable microenvironments for NSC growth and influence NSC behaviors (Li et al., 2013). Taken together, 3DG might have therapeutic potential for treating neurodegenerative diseases and neuronal disorders (Yang et al., 2016).

The proliferation of NSCs was promoted when cultured on 3DG (Li et al., 2013). NSC metabolism plays an important role in cell fate decisions, especially proliferation. Numerous studies have reported that the cross-talk between metabolic pathways and signaling pathways impacts the fate decision of NSCs (Kim et al., 2014). For example, the glycolysis pathway is regulated by FoxO3 to promote adult stem cell proliferation and differentiation (Yeo et al., 2013). The pentose phosphate pathway is regulated by FoxO3 and p53, which are necessary for proliferation, growth, the maintenance of the reduced glutathione level and the suppression of cellular oxidative stress by generating NADPH (Bensaad et al., 2006, 2009; Zhao et al., 2009). In addition, glutamine metabolism is affected by FoxO3, TSC/mTOR, HIF-1, and Sirtuin (Nicklin et al., 2009; Hensley et al., 2013; Son et al., 2013), and single-carbon metabolism and lipid metabolism are regulated by mTOR, HIF-1 and MYC during proliferation and neurogenesis (Metallo et al., 2011; Knobloch et al., 2013; Sun and Denko, 2014). ABC transporters and aminoacyl-tRNA synthesis are also involved in the metabolic activities of NSCs (Ibba and Soll, 2004; Lin et al., 2006). In turn, glutamine metabolism provides carbon for lipids and glutathione biosynthesis and nitrogen for nucleotide biosynthesis and thus regulates oxidative stress and promotes tumor proliferation (Wise and Thompson, 2010; Le et al., 2012). Proliferation of skeletal stem cells can be regulated through glutamine metabolism (Yu et al., 2019), and the proliferation of intestinal stem cells can be regulated by mitochondrial pyruvate metabolism (Schell et al., 2017).

Despite these previous studies, the underlying mechanism of how graphene systems influence the metabolism of NSCs is still poorly understood and requires more careful study, especially for 3DG. Understanding the interplay between metabolism, related metabolites, and enzymes has proven to be a critical task. In this study, the influence of 3DG on NSC proliferation and cell fate decision has beenexplored using gas chromatography-mass spectrometry (GC-MS)-based metabolomics techniques.

## Results

### Synthesis and Characterization of 3DG

**Figure 1:**
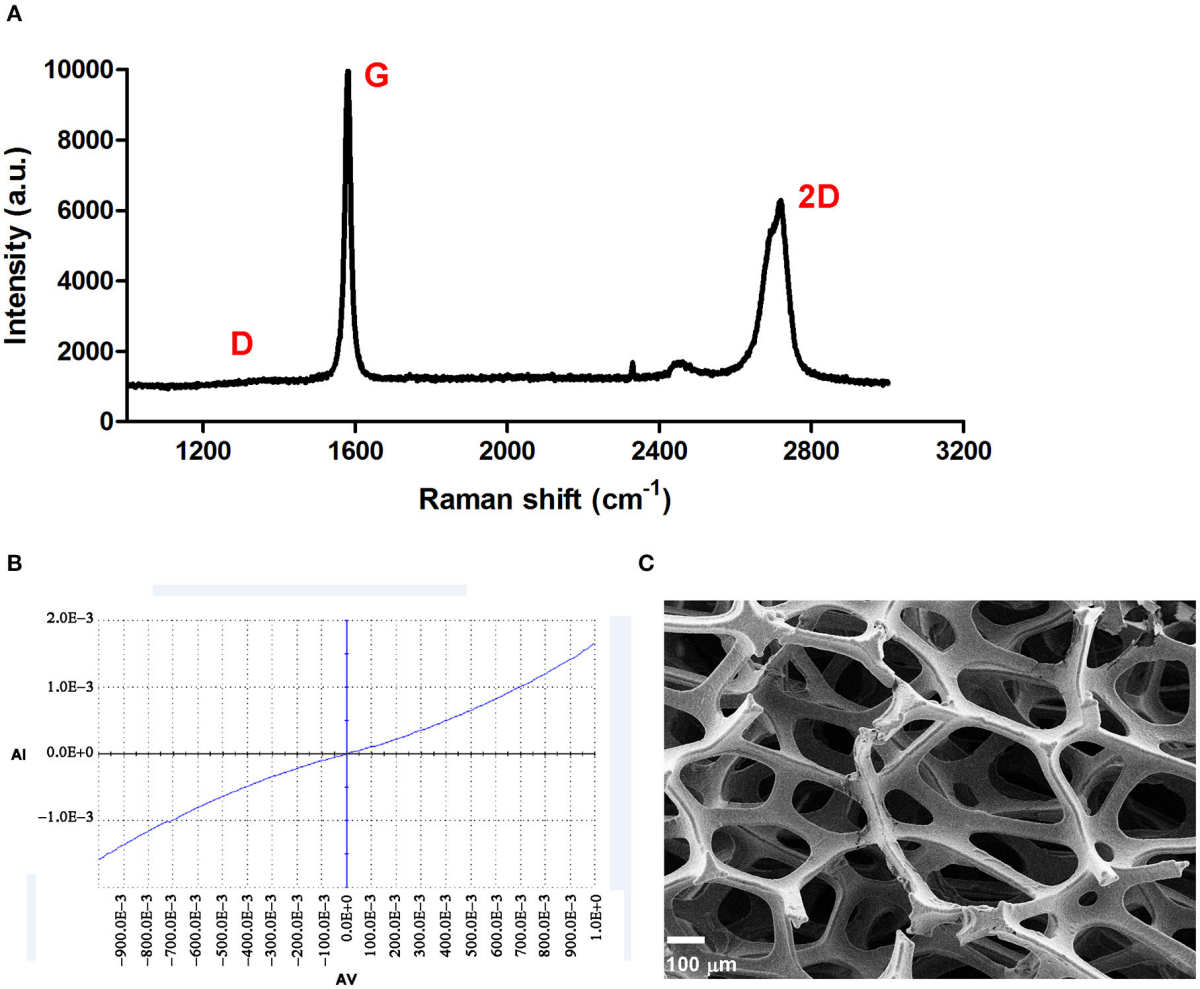
Characterization of 3DG. **(A)** Typical Raman spectrum of 3DG. **(B)** Current curve of 3DG. **(C)** SEM micrograph of 3DG. Scale bar = 100 μm.

The 3DG used in this study was synthesized as previously reported (Li et al., 2013). A typical Raman spectrum of 3DG was shown in Figure 1A, and the absence of a D band indicated that the 3DG was of high quality with few defects, while the intensity ratio between 2D and G bands and the shape of 2D band showed that the 3DG was made of only a few layers of graphene sheets (Ferrari et al., 2006). The I-V curve for 3DG is shown in Figure 1B and indicated excellent electrical conductivity (Figure 1B). The morphology of the 3DG was observed via scanning electron microscopy and showed a continuous and interconnected network with pores which can provide a three-dimensional scaffold to support NSCs attachment and growth, even it looked like a thin membrane. The porosity of 2DG and 3DG were determined to be 99.5 ± 0.2% and pore size of 3DG approximately 100–300 μm (Figure 1C).

### NSC Proliferation on 3DG

**Figure 2:**
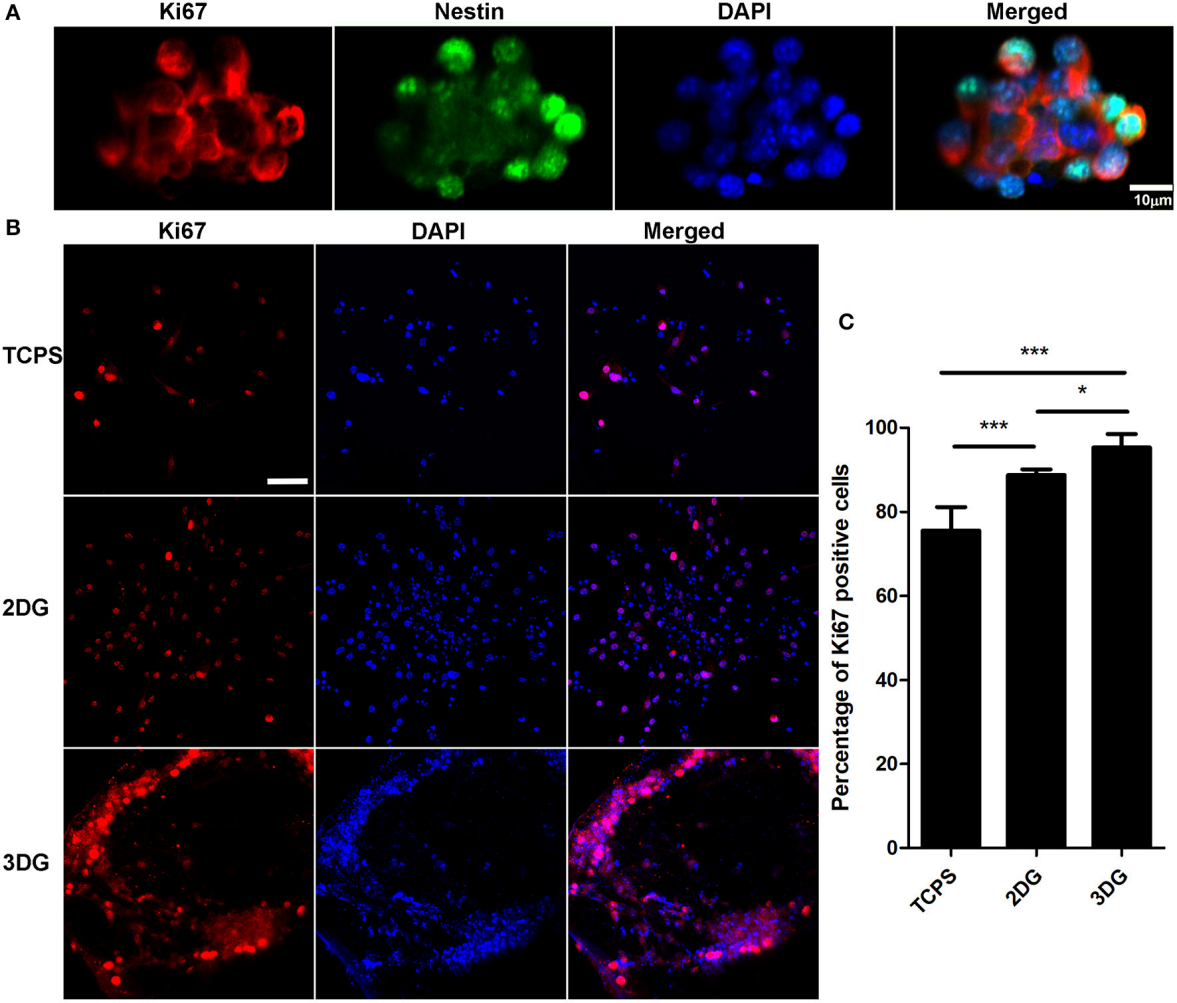
Proliferation of NSC. **(A)** Immunostaining image of NSC spheres, scale bar = 10 μm. **(B)** Proliferation detected after 5 days culture using Ki-67 immunolabeling, nuclei were labeled using DAPI. Scale bar = 50 μm. **(C)** Quantification data of **(B)** shows percentage of Ki-67 positive cells among three groups. *n* = 5, data were shown as mean ± SD, ^*^p < 0.05, ^***^ *p* < 0.001.

In order to purify and confirm NSCs, immunostaining with Nestin and Ki67 antibodies were performed (Figure 2A), neurospheres were collected and digested into single cells, after four generations, NSCs were seeded and cultured on tissue culture polystyrene (TCPS), 2D graphene film (2DG), and 3DG which coated with laminin. As a widely used biomolecule for NSC culture, laminin can produce a NSC-favorable surface to enhance cell adhesion. The proliferation ability of the NSCs on the different scaffolds was measured by immunostaining with Ki67, a cellular marker of proliferation (Sun and Kaufman, 2018) (Figures 2B,C). The number of cells and Ki67 positive cells were smaller in TCPS than 2DG[*F*_(2,14)_ = 35.06, *p* < 0.001], this might because graphene enhances neural stem cell proliferation ability. 3DG surface provided sufficient area for the attachment and growth of NSCs, and most NSCs on the surface of 3DG were Ki67 positive [*F*_(2,14)_ = 35.06, *p* < 0.05], which was consistency with previous reports (Kenry et al., 2016; Liu et al., 2016).

### Overall Metabolic Profiles of the NSCs

Using the GC-MS metabolomics approach, we found obvious differences between mouse NSCs grown on TCPS, 2DG, and 3DG for 5 days. We identified a total of 263 metabolites, including carboxylic acids, organic acids and their derivatives, amino acids and their derivatives, lipids, cofactors, prosthetic groups, and electron carriers as well as nucleotides and secondary metabolites (Supplementary Figure 1 and Data Sheet 1). These metabolites cover most of central metabolism and reflect the physiological status of NSCs.

**Figure 3:**
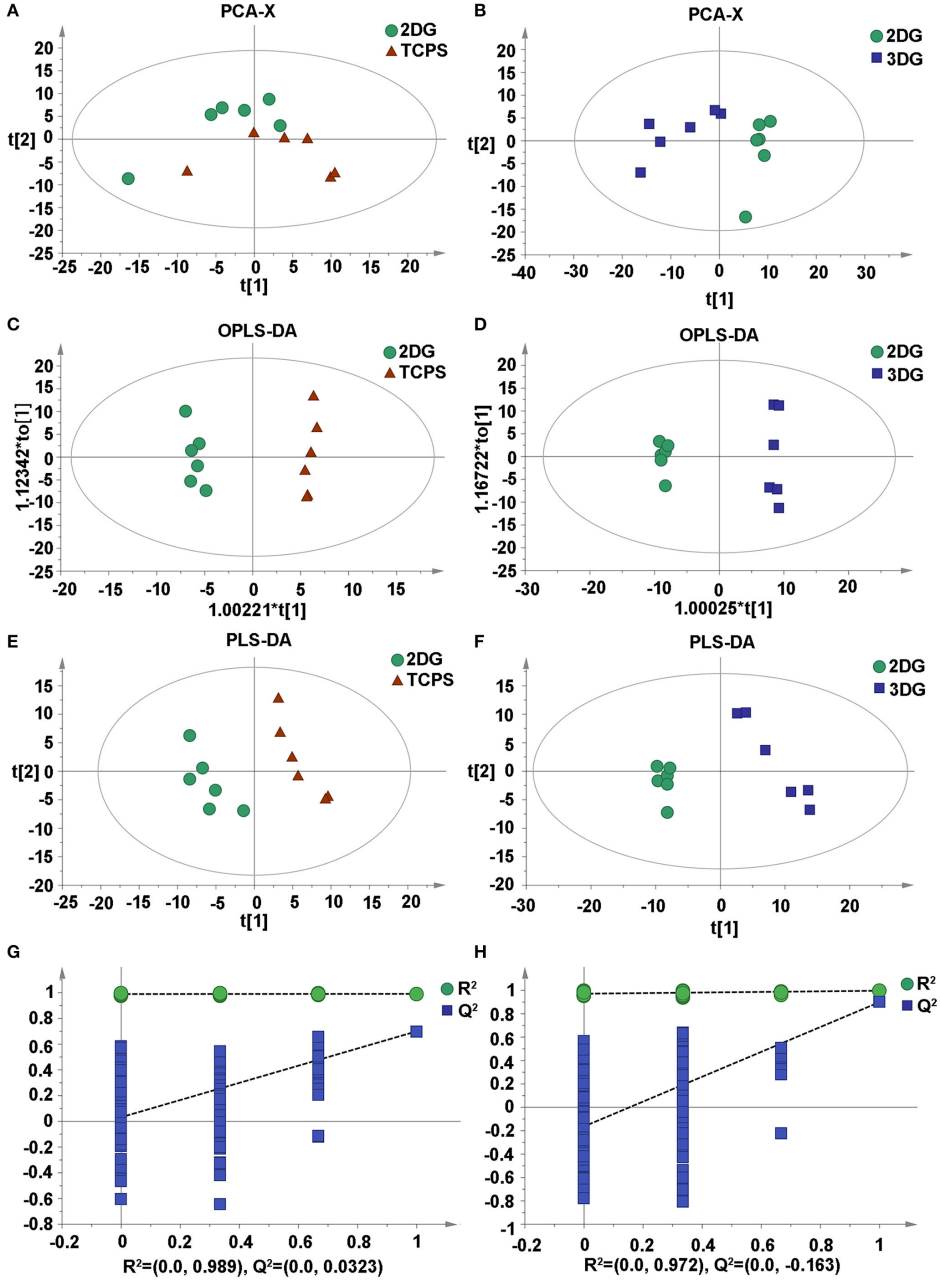
Multivariate statistical score graph among three groups. **(A)** PCA score plot of 2DG and TCPS. **(B)** PCA score plot of 3DG and 2DG. **(C)** OPLS-DA score plot of 2DG and TCPS. **(D)** OPLS-DA score plot of 3DG and 2DG. **(E)** PLS-DA score plot of 2DG and TCPS. **(F)** PLS-DA score plot of 3DG and 2DG. **(G)** Statistical validation with permutation analysis (200 times) of the corresponding PLS-DA model of 2DG and TCPS, R^2^ is the explained variance, and Q^2^ is the predictive ability of the model. **(H)** Statistical validation with permutation analysis (200 times) of the corresponding PLS-DA model of 3DG and 2DG, R^2^ is the explained variance, and Q^2^ is the predictive ability of the model.

An unsupervised principal component analysis (PCA) was used to observe the overall distribution of the samples and the stability of the overall analysis process. The PCA score plots showed classification trends between the 3DG, 2DG, and TCPS groups, with all of the observations falling within the Hoteling T^2^ (0.95) ellipse (2DG-TCPS: R^2^X = 0.43; Q^2^Y = 0.0498; 3DG-TCPS: R^2^X = 0.507; Q^2^Y = 0.176; 3DG-2DG: R^2^X = 0.549; Q^2^Y = 0.265). Subsequently, a supervised partial least-squares discriminant analysis (PLS-DA) model was used to identify the differences among NSCs culturing under the three conditions (2DG-TCPS: R^2^X = 0.5; R^2^Y = 0.991; Q^2^ = 0.857; *N* = 12), (3DG-TCPS: R^2^X = 0.498; R^2^Y = 0.996; Q^2^ = 0.966; *N* = 12), and (3DG-2DG: R^2^X = 0.339; R^2^Y = 0.936; Q^2^ = 0.969; *N* = 12). The PLS-DA model was also validated and passed the permutation test, which showed no observable over-fitting. In addition, we conducted an orthogonal partial least-squares discriminant analysis (OPLS-DA) on these data (Figure 3 and Supplementary Figure 2).

These results demonstrate that different growth conditions indeed induce significant reconfigurations among a large number of metabolic pathways. These changes in turn reflect differences in the NSCs' growth parameters, and thus it would be useful to understand the metabolic mechanism behind this phenotype variation. This is especially the case for the 3DG-2DG comparison, which has a larger difference compared to the 2DG-TCPS comparison, because this might indicate the importance of spatial contacts between cells, which are closer to the *in vivo* state in 3DG compared to 2DG.

### Distinctive Metabolomes of NSCs Cultured on 3DG, 2DG, and TCPS

NSCs produce different sets of metabolites when they are cultured on different materials. The levels of metabolites were considered significantly different if the variable influence on projection (VIP) value of the first principal component of the OPLS-DA was >1 and the *p*-value of the *t*-test was < 0.05. The relative abundance of 36 metabolites exhibited statistically significant differences between NSCs grown on 2DG and TCPS, with 8 metabolites higher in 2DG and 26 higher in TCPS. There were 93 differentially abundant metabolites between NSCs grown on 3DG and 2DG, with 37 metabolites being higher and 42 metabolites being lower in 3DG (Data Sheet 1). Among them, the most significantly up-regulated metabolites annotated in Table 1 were from the glucose degradation pathway (glucose-6-phosphate, gluconic acid, and D-glyceric acid) or essential amino acids (isoleucine, valine, phenylalanine, tyrosine, and lysine). All of these are needed for rapid cell growth and cell proliferation.

**Table 1:** The top 30 metabolites contributing significantly to discriminating cells grown on 2DG from those grown on TCPS and discriminating cells grown on 3DG from those grown on 2DG.

**Metabolites**	***p*-value**	**FC(2DG/TCPS)**	**Metabolites**	***p*-value**	**FC(3DG/2DG)**
Aconitic acid	1.07324E-06	^***^	Noradrenaline	2.57505E-09	^***^
Methyl-beta-D-galactopyranoside	4.56232E-05	0.317870954	Methyl phosphate	8.61821E-09	^***^
Gly-pro	0.000373616	3.45951521	Isoleucine	8.14022E-08	3.518754687
Phosphate	0.000813695	3.446777793	Valine	1.38383E-07	2.227047157
Adenosine 5-monophosphate	0.002511806	0.311194121	Serine	3.24336E-07	2.896413697
Beta-glycerophosphoric acid	0.004631138	0.663134866	Maleamate	3.73766E-07	2.062659789
Zymosterol	0.005497805	0.645471315	Glycerol	1.10385E-06	0.185467901
Threonine	0.005498206	0.788136334	D-glyceric acid	1.35641E-06	6.838118979
Glutamic acid	0.006031692	0.875420839	Glucose	1.94841E-06	0
5-Hydroxyindole-3-acetic acid	0.006584791	0.390133763	Hippuric acid	2.56832E-06	0
D-(glycerol 1-phosphate)	0.007013536	0.639306064	Tagatose	2.62655E-06	0.366839581
Asparagine	0.007221201	0.863352541	Phenylalanine	1.33697E-05	2.262626572
Glutamine	0.008681747	0.829487685	Fumaric acid	1.58222E-05	0.355499536
p-Anisic acid	0.010866232	2.610415225	Methionine	3.25786E-05	2.536990702
Hypoxanthine	0.012409871	0.786350146	Zymosterol	3.79166E-05	0.04548959
Lysine	0.01344286	0.830148494	Myo-inositol	4.79027E-05	0.097851995
Pyridoxine	0.015190631	0.850717833	D-(glycerol 1-phosphate)	5.27231E-05	0.123170735
4-Hydroxybenzyl cyanide	0.01574558	0.408363696	Melatonin	6.63317E-05	^***^
Methyl phosphate	0.016445895	0	Tyrosine	8.27556E-05	2.30350299
Arachidonic acid	0.016462751	0.494956435	Pyridoxine	0.000101939	1.901166986
Saccharopine	0.016550748	1.527539438	L-cysteine	0.000114856	0.123003722
Tartronic acid	0.017334697	0.4177656	Allose	0.000143555	2.421915203
Tyrosine	0.017829593	0.854202113	Tryptophan	0.000153858	3.055253667
3-Aminopropionitrile	0.01918978	12.84284424	Cholesterol	0.000160539	0.169891796
Phenylalanine	0.022176113	0.889029039	Lysine	0.000223344	3.370009385
Tryptophan	0.025824103	0.76103943	Gluconic acid	0.000239199	2.90495105
O-phosphonothreonine	0.026995702	0.327946302	O-methylthreonine	0.000344787	1.879317736
D-fructose 1,6-bisphosphate	0.029010308	0.687092159	Glucose-6-phosphate	0.000523658	2.397178129
Histidine	0.030446806	0.234203095	3-Cyanoalanine	0.000749964	1.444802925
Valine	0.032441598	0.914732445	O-phosphorylethanolamine	0.000770173	0.241073435

*** *Indicates that the metabolite was only found in cells grown on 2DG or 3DG*.

Different metabolites were compared among the three groups in volcano plots (Supplementary Figure 3), and the results indicated higher metabolic activities in NSCs grown on 3DG compared to those grown on 2DG and TCPS.

### Correlation Analysis Among Metabolites

**Figure 4:**
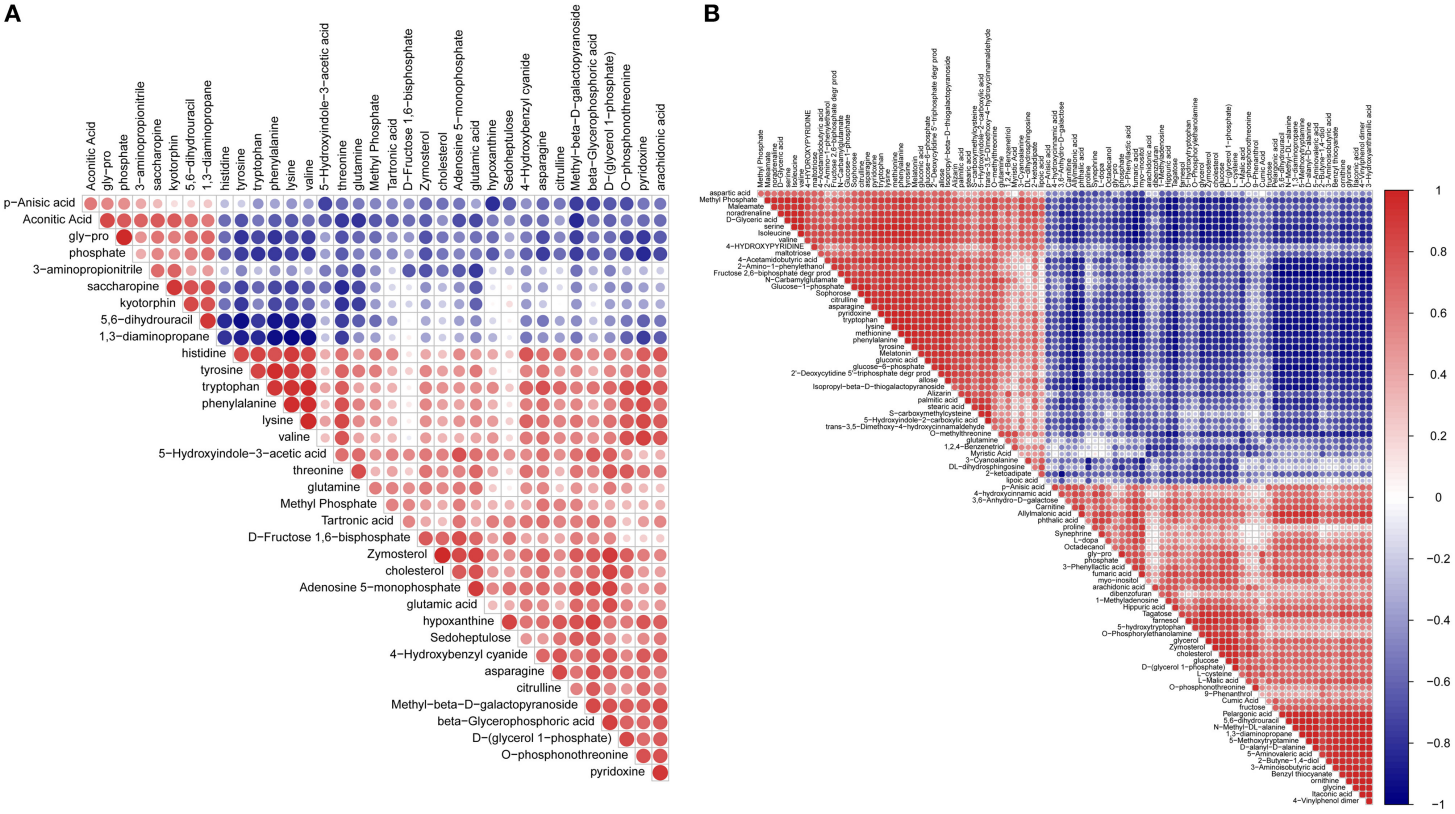
Metabolites-metabolites correlation analysis. Positive correlations are shown in red; negative correlations are shown in blue. **(A)** Metabolites-metabolites correlations of 2DG vs. TCPS. **(B)** Metabolites-metabolites correlations of 3DG vs. 2DG.

The matrix of correlation values provided more detailed information and a better overview of the relationships among related metabolites (Figure 4 and Data Sheet 2). A high positive correlation co-efficient means the enzymes producing the two metabolites likely function in the same pathway or have shared regulators in common. For instance, most of the highly correlated metabolites were between amino acids or between amino acids and carbohydrates, such as between tyrosine and valine or between serine and α-glucose-β-glucoside. Similarly, some lipids have very high correlations such as arachidonic acid and linoleic acid. High positive correlations between glucose and glucose-1-phosphate, glucose-1-phosphate and glucose-6-phosphate, asparagine and aspartic acid, and glutamic acid and glutamine are due to having the same substrates, enzymes, and pathways in their biosynthesis. A high negative correlation coefficient might reflect a competitive pathway or a pathway responsible for a different function.

### Differential Metabolite-Related Enzyme Changes

The metabolite concentration can be considered as a reflection of the integration of the *in vivo* enzyme activity along the growth time. A high abundance metabolite is probably associated with a high activity of the enzymes producing it as well as a low activity of enzyme consuming it. Therefore, here we define an enzyme activity score, which is obtained by normalizing the value of (substrate concentration—product concentration). The value of the score is associated with the direction and probability of the enzyme activity's response induced by change of micro-environment. The reaction ID is the Kyoto Encyclopedia of Genes and Genomes (KEGG) term. Compared to TCPS, 2DG induced changes in the activities of the following enzymes: α-aminoadipate semialdehyde synthase, dihydropyrimidine dehydrogenase, phenylalanine-4-hydroxylase, tyrosine 3-monooxygenase, thyroid peroxidase, pyrimidine-5′-phosphate oxidase, ornithine carbamoyl transferase, nitric oxide synthase, argininosuccinate synthase, pyridoxine kinase, L-amino acid oxidase, tyrosinase, and dihydropyrimidinase (Supplementary Table 1). Relative to 2DG, 3DG changed the activities of the following enzymes: ornithine carbamoyltransferase, indoleamine 2,3-dioxygenase, tryptophan 5-monooxygen enzyme, L-serine/L-threonine ammonia lyase, phosphoserine phosphatase, alanine-glyoxylate aminotransferase/serine-glyoxylate aminotransferase/serine-pyruvate transaminase, betaine-homocysteine S-methyltransferase, phenylalanine-4-hydroxylase, thyroid peroxidase, fatty acid synthase, pyridoxamine 5′-phosphate oxidase, tyrosine 3-monooxygenase, fatty acid synthase, pyridoxine kinase, long chain acyl-CoA synthetase, L-amino acid oxidase, inositol-1-phosphate synthase, glycine hydroxymethyltransferase, L-amino acid oxidase, S-adenosylmethionine synthetase, serine palmitoyltransferase, cystathionine β-synthase, serine racemase, tyrosinase, L-amino acid oxidase, aromatic-L-amino acid/L-tryptophan decarboxylase, mono-oxidation nitrogen synthase, and arginine succinate synthetase (Supplementary Table 2). In comparing the 2DG and TCPS groups, 32 enzymes had scores >0 and 37 enzymes had scores <0. In comparing the 3DG and 2DG groups, 58 enzymes had scores >0 and 56 enzymes had scores lower than 0. This suggested that more enzyme activities were changed in the 3DG group. In addition, when comparing Supplementary Table 1 with Supplementary Table 2, it can be seen that many enzymes had increased activity in NSCs grown on 3DG. These results indicate increased metabolic activity in cells grown on 3DG, which is consistent with increased NSC proliferation when grown on 3DG.

### Metabolic Pathway Analysis

**Figure 5:**
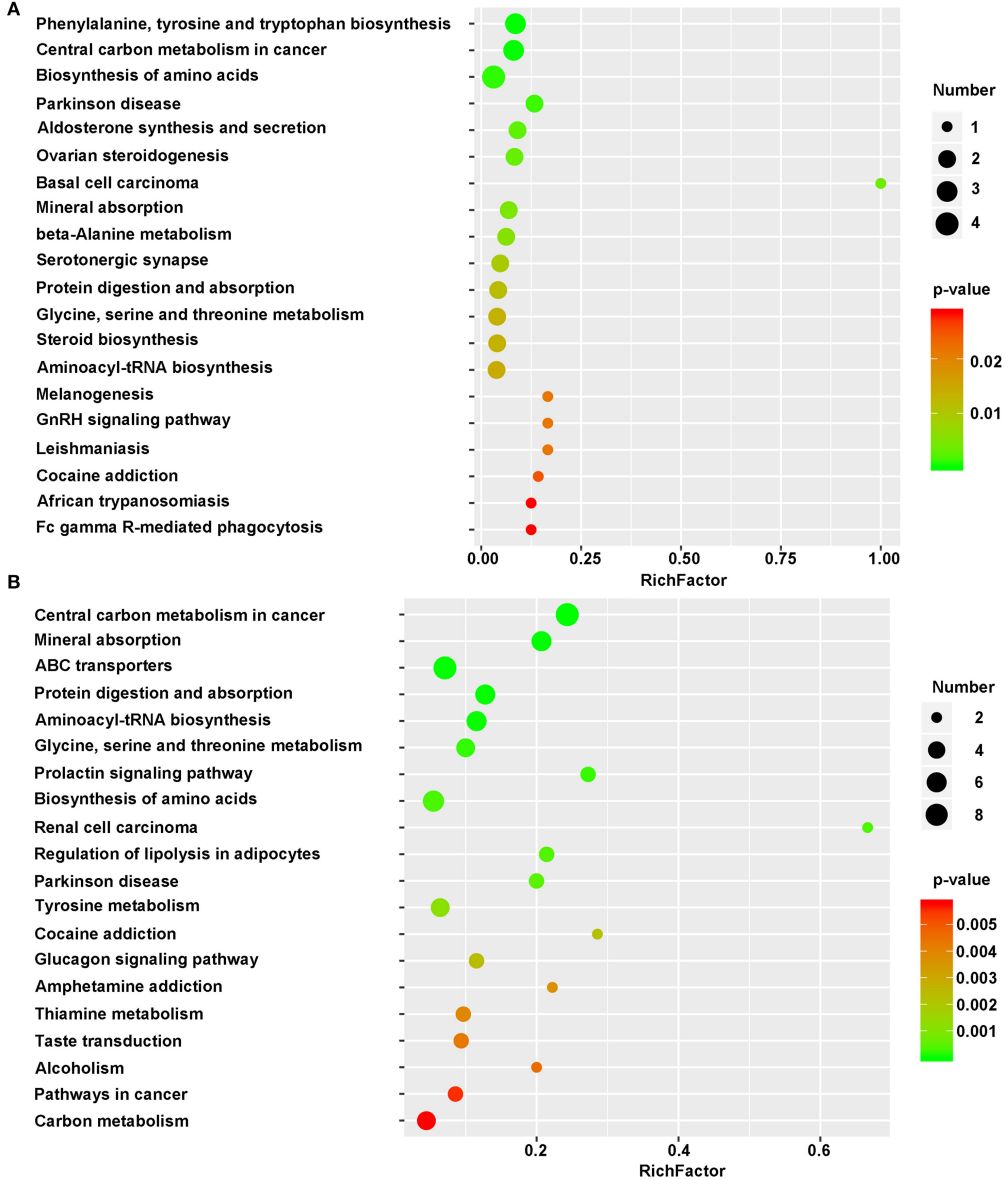
Bubble diagram of differential metabolic pathways. One bubble represent one metabolic pathway. Top 20 pathways were list in left side. Numbers of involved metabolites and the *p*-value were list on right side. **(A)** Bubble diagram of 2DG vs. TCPS. **(B)** Bubble diagram of 3DG vs. 2DG. X-axis represent rich factor. This figure was drawn using R language.

By concatenating the differentially regulated enzymes and metabolites, we identified the altered metabolic pathways in cells grown on the different substrates. The identified metabolites were mapped as defined by KEGG. The top 20 KEGG metabolic pathways and the pathway IDs were listed in Supplementary Table 3. Between the NSCs grown on 2DG and those grown on TCPS, some significantly altered metabolites belong to Phenylalanine, tyrosine, and tryptophan biosynthesis (mmu00400), central carbon metabolism in cancer (mmu05230), Biosynthesis of amino acids (mmu01230), Parkinson disease (mmu05012), Aldosterone synthesis and secretion (mmu04925), Ovarian steroidogenesis (mmu04913), Basal cell carcinoma (mmu05217), Mineral absorption (mmu04978), Beta-alanine metabolism (mmu00410), Serotonergic synapse (mmu04726), Protein digestion and absorption (mmu04974), Glycine, serine, and threonine metabolism (mmu00260), Steroid biosynthesis (mmu00100), and Aminoacyl-tRNA biosynthesis (mmu00970) (Figure 5A).

However, a smaller number of metabolic pathways were altered when comparing NSCs grown on 2DG to those grown on TCPS than when comparing NSCs grown on 3DG to those grown on 2DG. Figure 5 shows that the rich factor of the 3DG/2DG comparison is much higher than that of the 2DG/TCPS comparison. This is also the case for *p*-value. The shift from 2DG to 3DG induced regulation of a large number of pathways, including Central carbon metabolism in cancer (mmu05230), Mineral absorption (mmu04978), ABC transporters (mmu02010), Protein digestion and absorption (mmu04974), Aminoacyl-tRNA biosynthesis (mmu00970), Glycine, serine, and threonine metabolism (mmu00260), Biosynthesis of amino acids (mmu01230), and Tyrosine metabolism (mmu00350) (Figure 5B). The ABC transporter pathway might be responsible for increased levels of essential amino acids seen in NSCs grown on 3DG, and increased activity of the aminoacyl-tRNA biosynthesis pathway is consistent with the elevated protein synthesis needed due to the high growth rate of these cells.

## Discussion

As a carbon-based nanomaterial, graphene has excellent physical and chemical properties. Since its discovery in 2004 (Geim and Novoselov, 2007), it has not only been a hot topic in materials science and condensed matter physics, but has also evoked interest in the biomedical field. For example, graphene has been used for drug transport, stem cell engineering, cell imaging, and tumor therapy (Zhu et al., 2010). Graphene oxide attracted much attention due to the larger surface and oxygen groups, It has been demonstrated that graphene oxide can be used in drug delivery of cancer therapy (Liu et al., 2018). In addition, study has reported that graphene oxide can be used in cell imaging without any additional fluorescent protein (Cheng et al., 2018).

The main function of NSCs is to serve as a cellular reserve to participate in the repair of nervous system damage or to replace dead cells (Forsberg et al., 2017). The hippocampus is the part of the brain involved in bodily sensations, learning, memory, and homeostasis, and hippocampal NSCs have been used for decades as a typical model of the nervous system (Hattiangady and Shetty, 2012). Hippocampal NSCs hold the potential to treat or cure many neurological diseases and injuries. Particularly neurodegenerative diseases, like AD, PD, spinal cord injuries, and cerebral strokes (Taupin, 2011). Uncovering the underlying mechanism of the proliferation and differentiation of NSCs, especially regulated by metabolomics of the cells.

Metabolomics plays an increasingly prominent role in biomarker identification in various neurodegenerative disorders such as Parkinson's disease, Huntington disease, schizophrenia, and Batten disease (Pears et al., 2010; Oresic et al., 2011; Hatano et al., 2016; Skene et al., 2017). Parkinson's disease is characterized by the gradually develop of resting tremor, bradykinesia, and postural etc. Even the exact mechanism is not known, the abnormal metabolites may be underlined with several proteins expression. For example, the change of amino acids expression has been reported in past decades. Reduction of alanine, arginine, lysine and methionine, increasing of glycine, valine, and aspartate are related with the Parkinson's disease progression (Iwasaki et al., 1992; Jimenez-Jimenez et al., 1996; Mally et al., 1997).

The methodology of this study included a standardized non-targeted GC-MS-based metabolic profiling approach combined with statistical analysis. We found significant differences in the metabolomes between cells grown on TCPS, 2DG, and 3DG, and differential enrichment analysis showed significant changes in metabolic pathways between NSCs grown on 2DG and 3DG, such as the ABC transporter system, mineral uptake, protein digestion and absorption, and aminoacyl-tRNA biosynthesis. Protein digestion and absorption are highly relevant for the biosynthesis of amino acids, and ATP-binding (ABC) transporters form one of the largest known families of proteins that combine ATP hydrolysis with active transport of multiple substrates such as ions, sugars, amino acids lipids, sterols, peptides, proteins, and drugs. Together, protein digestion and absorption, amino acid biosynthesis, and the ABC transporter pathway provide both the non-essential and essential amino acids needed for cell proliferation and differentiation (Besingi and Clark, 2015; Price et al., 2018).

The correlation of metabolomics reconfiguration with cell proliferation is very interesting. For example, zymosterol, which is essential for cholesterol synthesis shown lower expression in TCPS group when compared with 2DG and 3DG groups. The proliferation of cells was inhibited when zymosterol and cholesterol were reduced (Urbina et al., 1997; Wang et al., 2019). T cell proliferation was inhibited by macrophage Tryptophan catabolism, therefore, the reduction of Tryptophan in 2DG when compared with TCPS consistent with our finding. Liver cancer development can be attenuated by inhibiting glycerol synthesis (Capiglioni et al., 2018), The reduction of glycerol also match with graphene enhancing NSCs proliferation. Glucose-6-phosphate promotes the proliferation of fibroblast-like synoviocytes (Zong et al., 2015). It is reasonable since high glucose-6-phosphate is associating with high activity glucose metabolism, which provides more energy during proliferation. The proliferation-related metabolites show more significant change when NSCs are grown on 3DG compared to 2DG or TCPS (Table 1).

Minerals are one of the five essential nutrients needed to sustain life (Ali et al., 2014). For example, calcium plays many roles in the body, and it is the main component of bones and is an intracellular messenger of muscle contraction/relaxation, neural networks, the immune system, and secretory cells. In addition to this, iron, copper, and other metal ions are required as cofactors in redox reactions and for the formation of hemoglobin and myoglobin for oxygen binding/transport (Novak Kujundzic et al., 2014). Many enzymes also require specific metal atoms in order to perform their catalytic functions (Copley, 2003).

It is also worth noting that 3DG and 2DG induce the metabolic pathways involved in Parkinson's disease in cultured NSCs. Abnormal amino-acid metabolism include Phenylalanine, tyrosine, tryptophan, Glycine, Serine and Threonine metabolism are involved in Parkinson's disease development, biosynthesis of amino-acid as well (Zhao et al., 2018). In general, the primary metabolic pathways enriched in Parkinson's disease were, tyrosine biosynthesis, glycerol phospholipid metabolism and bile acid biosynthesis. Recent study also shows abnormal cholesterol level in the Parkinson's patients (Luan et al., 2015). As primary energy resources, lipid and glucose metabolism provide most of energy in physiological condition. ABC transporters mediate the export of numerous substances, for example drugs and hydrophobic molecules. The relationship between ANC transporter and Parkinson's disease was based on the function of ABC transporter, the abnormal ABC transporter expression in PD has been reported. In this study, ABC transporter enriched when compared 2DG with 3DG. Further study also need to investigate if ABC transporter can be a candidate therapy.

In addition, many metabolite changes here show relevance to Parkinson' disease (Table 1). the reduction of tyrosine which involved in Parkinson's disease was found in 2DG group, when compared to TCPS group. The increasing of L-DOPA also found in 3DG group when compared to 2DG group. In recent years, researchers found that the reduction of tyrosine or the increasing of L-DOPA might be the candidate therapy of Parkinson's disease (Jankovic, 2005; Tanabe et al., 2014). The pathologic activities in Parkinson' disease can be mediated by asparagine endopeptidase (Zhang et al., 2017). The reduction of the glutamate metabolizing enzyme glutamine synthase activity may lead to redundant glutamate in Parkinson's patients (Zipp et al., 1998). arachidonic acid, which upregulated in Parkinson's disease mice, may contribute to the symptoms and pathology (Lee et al., 2010). Phenylalanine hydroxylase as a biomarker of Parkinson's disease also play important role in pathology development, even the detail mechanism is not well-known (Steventon and Mitchell, 2018). High level of serum cholesterol reduce the Parkinson's disease risk (Rozani et al., 2018). The data suggested a potential association between graphene and pathways involved in Parkinson's disease, and more research is required to get a better understanding.

In addition to understanding the function of an enzyme group, the roles of individual enzymes can be discussed in detail. Ornithine transcarbamylase is an aminotransferase and is mainly involved in the urea cycle (Nagasaka et al., 2013). Deficiency in the enzyme results in hypotonia, seizures, mental retardation, and hyperammonemia, which refers to elevated levels of ammonia in the blood that can cause irreversible brain damage if not treated early. The high expression of ornithine transcarbamylase in NSCs grown on 3DG might be useful in recycling the ammonia produced as a by-product of rapid proliferation. As such, 3DG can become a good drug carrier for those diseases due to its extra capacity to induce this enzyme's expression. Nitric oxide synthase is an oxidoreductase involved in various biological processes such as neurotransmission, amino acid metabolism, environmental signaling pathways, catabolism (Li et al., 2007; Naglah et al., 2016), the endocrine system, and smooth muscle motor control (Adams and Bronner-Fraser, 2009; Hillard, 2015), neuronal damages, neurodegeneration, and NSCs proliferation (Chong et al., 2018). L-amino acid oxidase is involved in the biosynthesis and metabolism of amino acids (Niedermann and Lerch, 1991), and we found that 3DG causes down-regulation of this enzyme in NSCs. Phenylalanine-4-hydroxylase is involved in phenylalanine metabolism and in the phenylalanine, tyrosine, tryptophan, and folate biosynthesis pathways (Lin et al., 2014). Tyrosine 3-monooxygenase is an oxidoreductase that is involved in the metabolism of amino acids, cofactors, and vitamins and in the biosynthesis of other secondary metabolites, as well as the redundant dopamine in the nervous system (Otten et al., 1974). Tyrosinase is involved in amino acid metabolism and in the biosynthesis of other secondary metabolites that affect signaling pathways and cell growth and development (Wu, 2010). Thyroid peroxidase is involved in the regulation of amino acid metabolism and cellular proliferation in thyroid follicular tumors (Garcia et al., 1998). Indole amine 2, 3-dioxygenase is mainly involved in the metabolism of tryptophan which is a factor to inhibit cellular proliferation of T-lymphocyte (Menta et al., 2014). Aromatic-L-amino acid/L-tryptophan decarboxylase is a lytic enzyme that cleaves carbon-carbon groups, participates in the synthesis of amino acids and other secondary metabolites, and plays a role in dopamine synapses in the nervous system and in the processes of cell development and growth (Murali et al., 2003; Kalb et al., 2016). Tryptophan 5-monooxygenase is involved in the metabolism of amino acids, co-factors, and vitamins and plays a role in the physiological activities of serotonin synapses in the nervous system (Fujisawa and Nakata, 1987). Arginine succinate synthetase is a ligase that is involved in amino acid metabolism, signaling pathways, and cell growth processes in cardiovascular diseases (Valeev et al., 2016). Several reactions in vitamin B_6_ metabolic pathway were catalyzed by Pyridoxamine 5'-phosphate oxidase and pyridoxine kinase which contribute to neurotransmitter and amino acid metabolism (Pandey et al., 2014; Huang et al., 2016; Ramos et al., 2017; Kim et al., 2018). In this study, the above enzyme scores in the 3DG-2DG comparison were higher than in the 2DG-TCPS comparison which also indicate the 3DG can enhance the proliferation of NSCs.

In summary, our results show that 3DG induces greater NSC proliferation compared to 2DG and TCPS. 3DG can not only mimic the *in vivo* environment very well, but can promote mineral absorption, protein digestion and absorption, biosynthesis of amino acids, and ABC transporter activity. Together, these properties allow for greater amino acid incorporation and enhanced glucose metabolism, which is likely the driving force for faster NSC growth on 3DG. In addition, our data show some correlation between the Parkinson's pathway and graphene, which need further study. Our findings suggest the possibility to regulate NSC cell fate through the regulation of metabolism and that 3DG might provide a powerful platform for investigating NSC proliferation and differentiation.

## Materials and Methods

### Chemicals

All chemicals and solvents were analytical or HPLC grade. Water, methanol, acetonitrile, pyridine, n-hexane, methoxylamine hydrochloride (97%), and N, O-bis (trimethylsilyl) trifluoroacetamide (BSTFA) with 1% trimethylchlorosilane (TMCS) were purchased from CNW Technologies GmbH (Düsseldorf, Germany). Trichloromethane was from Shanghai Chemical Reagent Co., Ltd. (Shanghai, China). L-2-chlorophenylalanine was from Shanghai Hengchuang Bio-technology Co., Ltd. (Shanghai, China).

### Characterization of 2DG and 3DG

The crystallinity and the number of the layers present within the graphene were determined using lamRAM (HR800, HORIBA, France). The surface chemistry was examined using XPS (Axis Ultra DLD, Kratos, UK) with an Al K α X-ray source operated at 40 eV.

### Graphene Synthesis and NSC Culture

For 2DGs, Cu foils were heated to 950°C, then annealed under H_2_ and Ar gases for 5 min, followed by exposure to CH_4_ and H_2_ for 5 min. Finally, substrates were cooled down to room temperature under H_2_ and Ar gases. The 3DGs were synthesized by similar method with Ni foam as template. 2DG and 3DG were firstly soaked in FeCl_3_ (1 M) solution for 72 h at room temperature. Then 3DG and 2DG were rinsed sequentially with 1 M, 0.1 M, and 0.01 M HCl solutions, followed by rinsing with running water for 72 h to remove the etching agents. Before using, 2DG and 3DG were sterilized with UV light and 70% alcohol and then clean with phosphate-buffered saline three times. NSCs were isolated and purified from the hippocampus of embryonic day 18.5 FVB mice. The hippocampus was dissected out and cells were dissociated with Accutase (Gibco, A11105) for 20 min at 37°C and then washed twice with 1 × PBS and then suspended in proliferation culture medium containing DMEM-F12 with EGF (20 ng/mL, R&D Systems, USA), 2% B27 supplement (Life Technologies, USA), FGF (20 ng/mL, R&D Systems, USA) and 1% penicillin-streptomycin (P/S, Sigma). Tissues were gently triturated using a pipette, and single cells were purified with a 40 μm filter (Corning). Then cells were transferred into sterile cell culture flask, cultured with 2 ml proliferation medium at 37°C with 5%CO_2_. After four generations, neuron spheres were collected and then digested into single cells. Before seeding NSCs, the graphene substrates and TCPS was coated with laminin (20 μg/mL, 37°C, 4 h) in six well plate (Greiner Bio-One, 657160). Three times wash was performed using 1x PBS to remove redundant laminin. NSCs were seeded at around 4 × 10^4^ cells/ml, 2 ml proliferation medium in total, then cultured for 5 days. All animal procedures were performed according to protocols approved by the Animal Care and Use Committee of Southeast University and were consistent with the National Institutes of Health Guide for the Care and Use of Laboratory Animals. All efforts were made to minimize the number of animals used and to prevent their suffering.

### Immunostaining for NSCs

After culturing for 5 days, NSCs were fixed with 4% paraformaldehyde for 30 min at room temperature and permeabilized with 0.5% Triton X-100 for 1 h. NSC spheres were stained with Nestin antibody (Beyotime Biotechnology, AN205), proliferation of NSCs was detected using the anti-Ki67 (Abcam, ab15580) antibody, which was incubated with the cells overnight at 4°C. DAPI was used to label NSC nuclei after the sample was washed three times with PBST (1 × PBS, pH 7.2, with 0.1% Triton X-100). Secondary antibody (Abcam, ab150075) was incubated for 1 h at room temperature. Finally, the NSCs were mounted on glass slides with Fluoromount-G mounting medium, and images were acquired on a Zeiss LSM 700 confocal microscope.

### Sample Preparation for GC-MS Metabolomics Analysis

NSCs were washed twice with 1 × PBS before being frozen in liquid nitrogen. The culture dishes were filled with 80% methanol, and the cells were collected into 2 mL Eppendorf tubes. A total volume of 20 μL of 2-chloro-l-phenylalanine (0.3 mg/mL) dissolved in methanol was added as the internal standard, and 200 μL of methanol was added to each sample, which were then transferred to a 2 mL glass vial. A total volume of 200 μL chloroform was added to each sample and mixed with a pipette. The cells were lysed in an ultrasonic homogenizer for 6 min at 500 W. Each sample was transferred to a 1.5 mL Eppendorf tube then dispersed by ultrasonication for 20 min in an ice water bath. The sample was centrifuged for 10 min, 12,000 g, 4°C. Over a 0.22 μm organic phase film, 800 μL of supernatant in a glass vial was freeze dried in a concentrating centrifugal dryer. A total volume of 80 μL of 15 mg/mL methoxylamine hydrochloride in pyridine was added, and the mixture was vortexed vigorously for 2 min and incubated at 37°C for 90 min. Finally, 80 μL of BSTFA (with 1% TMCS) and 20 μL n-hexane were added to the mixture, which was vortexed vigorously for 2 min and then derivatized at 70°C for 60 min. The samples were equilibrated to ambient temperature for 30 min before GC-MS analysis.

### GC-MS Analysis

The derivatized samples were analyzed on an Agilent 7890B gas chromatography system coupled to an Agilent 5977A MSD system (Agilent J & W Scientific, Folsom, CA, USA). A DB-5MS fused-silica capillary column (30 m × 0.25 mm × 0.25 μm, Agilent) was used to separate the derivatives. Helium (>99.999% purity) was used as the carrier gas at a constant flow rate of 1 mL/min through the column. The injector temperature was maintained at 260°C, and the injection volume was 2 μL by splitles mode. The initial oven temperature was 90°C, and this was ramped to 180°C at a rate of 10°C/min, to 240°C at a rate of 5°C/min, to 290°C at a rate of 25°C/min, and finally held at 290°C for 11 min. The temperature of the MS quadrupole was set to 150°C, and the ion source (electron impact) was set to 230°C. The collision energy was 70 eV. Mass data were acquired in full-scan mode (m/z 50–450), and the solvent delay time was set to 5 min.

The quality controls (QCs) were injected at regular intervals (every 9 samples) throughout the analytical run to provide a set of data from which repeatability could be assessed.

### Data Preprocessing and Statistical Analysis

The acquired MS data from GC-MS were analyzed with Chroma TOF software (v 4.34, LECO, St. Joseph, MI). The metabolites were identified using the NIST and Fiehn database, which is linked to the Chroma TOF software. Briefly, after alignment with the Statistic Compare component of the software, a CSV file was obtained with three-dimensional data sets including sample information, peak names, retention times-m/z, and peak intensities. There were a total of 552 detectable peaks in the GC-MS data from the samples, and the internal standard was used for data quality control (reproducibility). After internal standards and any known false-positive peaks—such as peaks caused by noise, column bleed, and the BSTFA derivatization procedure—were removed from the data set and the peaks from the same metabolites were combined, there were 263 detectable metabolites.

The resulting data were normalized to the total peak area of each sample, multiplied by 10,000, transformed by log2 in Excel 2007 (Microsoft, USA), and imported into SIMCA (version 14.0, Umetrics, Umeå, Sweden), where PCA, PLS-DA, and OPLS-DA were performed. The Hotelling's T^2^ region, shown as an ellipse in the score plots of the models, defines the 95% confidence interval of the modeled variation. The quality of the models is described by the R^2^X or R^2^Y and Q^2^ values. R^2^X and R^2^Y are defined as the proportions of variance in the data explained by the models and indicate the goodness of fit. Q^2^ is defined as the proportion of variance in the data predicted by the model and indicates predictability as calculated by a cross-validation procedure. A default seven-round cross-validation in SIMCA was performed throughout to determine the optimal number of principal components and to avoid model overfitting. The OPLS-DA models were also validated by a permutation analysis (200 times). The Ki67 positive cells among these groups were quantified and statistic analyzed, *p*-values from One Way ANOVA with *post-hoc* test.

### Identification of Differential Metabolites

The differential metabolites were selected on the basis of the combination of a statistically significant threshold of VIP values obtained from the OPLS-DA model and *p*-values from a two-tailed Student's *t*-test on the normalized peak areas, where metabolites with VIP values larger than 1.0 and *p* < 0.05 were included.

### KEGG Pathway Analysis of Identified Differential Metabolites

The identified differential metabolites were mapped onto the KEGG database with the Omics Bean software (http://www.geneforhealth.com) for KEGG pathways analysis.

## Data Availability Statement

All datasets generated for this study are included in the article/Supplementary Material.

## Ethics Statement

The animal study was reviewed and approved by the Animal Care and Use Committee of Southeast University.

## Author Contributions

QF, YZ, and RC conceived and designed the experiments. QF, YZ, XC, HL, and WZ performed the experiments. LC, ZZ, MT, WL, TS, HW, and TW analysis data. QF, TS, YZ, TW, and RC wrote the manuscript.

### Conflict of Interest

XC was employed by company Hangzhou Rongze Biotechnology Co. Ltd. The remaining authors declare that the research was conducted in the absence of any commercial or financial relationships that could be construed as a potential conflict of interest.

## Supplementary Material

Supplementary Figure 1 Total ion chromatogram from neural stem cells cultured within different systems using GC-MS. **(A)** total ion chromatogram from NSC cultured on TCPS. **(B)** Total ion chromatogram from NSC cultured on 2D graphene system. **(C)** Total ion chromatogram from NSC cultured in 3D graphene system. **(D)** Total ion chromatogram from QC sample.

Supplementary Figure 2 Multivariate statistical score graph among three groups. **(A)** PCA score plot of 3DG and TCPS. **(B)** OPLS-DA score plot of 3DG and TCPS. **(C)** PLS-DA score plot of 3DG and TCPS. **(D)** Statistical validation with permutation analysis (200 times) of the corresponding PLS-DA model of 3DG and TCPS, R^2^ is the explained variance, and Q^2^ is the predictive ability of the model.

Supplementary Figure 3 volcano map of 3D graphene, 2D graphene and TCPS. **(A)** 2DG vs. TCPS. **(B)** 3DG vs. 2DG. **(C)** 3DG vs. TCPS. up regulation (red), down regulation (green). The horizontal axis is log2 (Fold change), and the vertical axis is –log10 (*P*-value), each dot represents a gene, and the two lines parallel to the Y axis are X = 1 and X = −1, respectively. The point on the right side of X = 1 is a gene that is up-regulated by a factor of two or more, and the point on the left side of X = −1 is a gene that is down-regulated by a factor of two or more. At the same time, there is a dotted line Y = –log10 (0.05) parallel to the X axis, and a point above the dotted line indicates a gene with a significance *P* < 0.05. Significantly differentially expressed genes with >2-fold up-regulated genes are marked in red and down-regulated genes are marked in blue. In addition, the difference significance is <2 times and is marked with gray. Moreover, the farther away from the distance between the two axes of X = 1 and X = −1, the greater the variation of the gene represented by this point between the two groups. Through the volcano map, we can clearly see the difference in metabolites of neural stem cells cultured from three different materials. Compared with the 3DG and 2DG groups and the 3DG and TCPS groups, the significant metabolites were significantly better than the 2DG and more than TCPS groups.

Supplementary Figure 4 **(A)** Metabolites-metabolites correlations of 3DG vs. TCPS. **(B)** Bubble diagram of differential metabolic pathways in 3DG vs. TCPS group.

Supplementary Table 1 Changes in metabolite-related enzymes in two-dimensional graphene and ordinary slides.

Supplementary Table 2 Changes in differential metabolite-related enzymes in three-dimensional graphene and two-dimensional graphene groups.

Supplementary Table 3 Metabolic pathways and the KEGG ID in this study.

Datasheet 1 Metabolites change in three comparable groups.

Datasheet 2 Metabolites-metabolites correlations in three comparable groups.
