# Supplementary figures and images for: Three-Dimensional Graphene Enhances Neural Stem Cell Proliferation Through Metabolic Regulation
Source: Front Bioeng Biotechnol. 2020 Jan 8;7:436. doi: 10.3389/fbioe.2019.00436 (PMC6961593; doi:10.3389/fbioe.2019.00436)

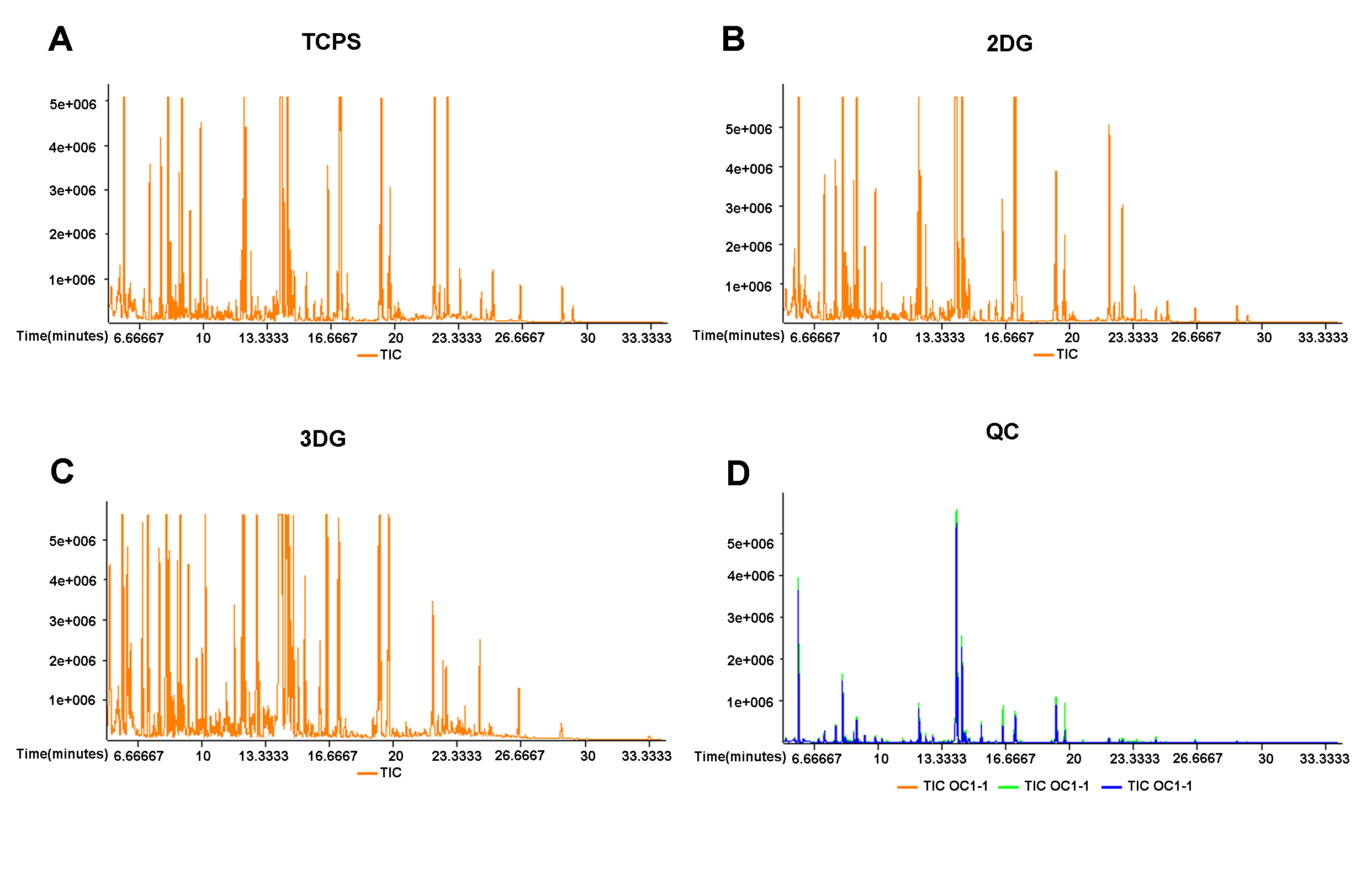

Supplement: Supplementary Figure 1 — Total ion chromatogram from neural stem cells cultured within different systems using GC-MS. (A) total ion chromatogram from NSC cultured on TCPS. (B) Total ion chromatogram from NSC cultured on 2D graphene system. (C) Total ion chromatogram from NSC cultured in 3D graphene system. (D) Total ion chromatogram from QC sample. [file Image_1.TIF]

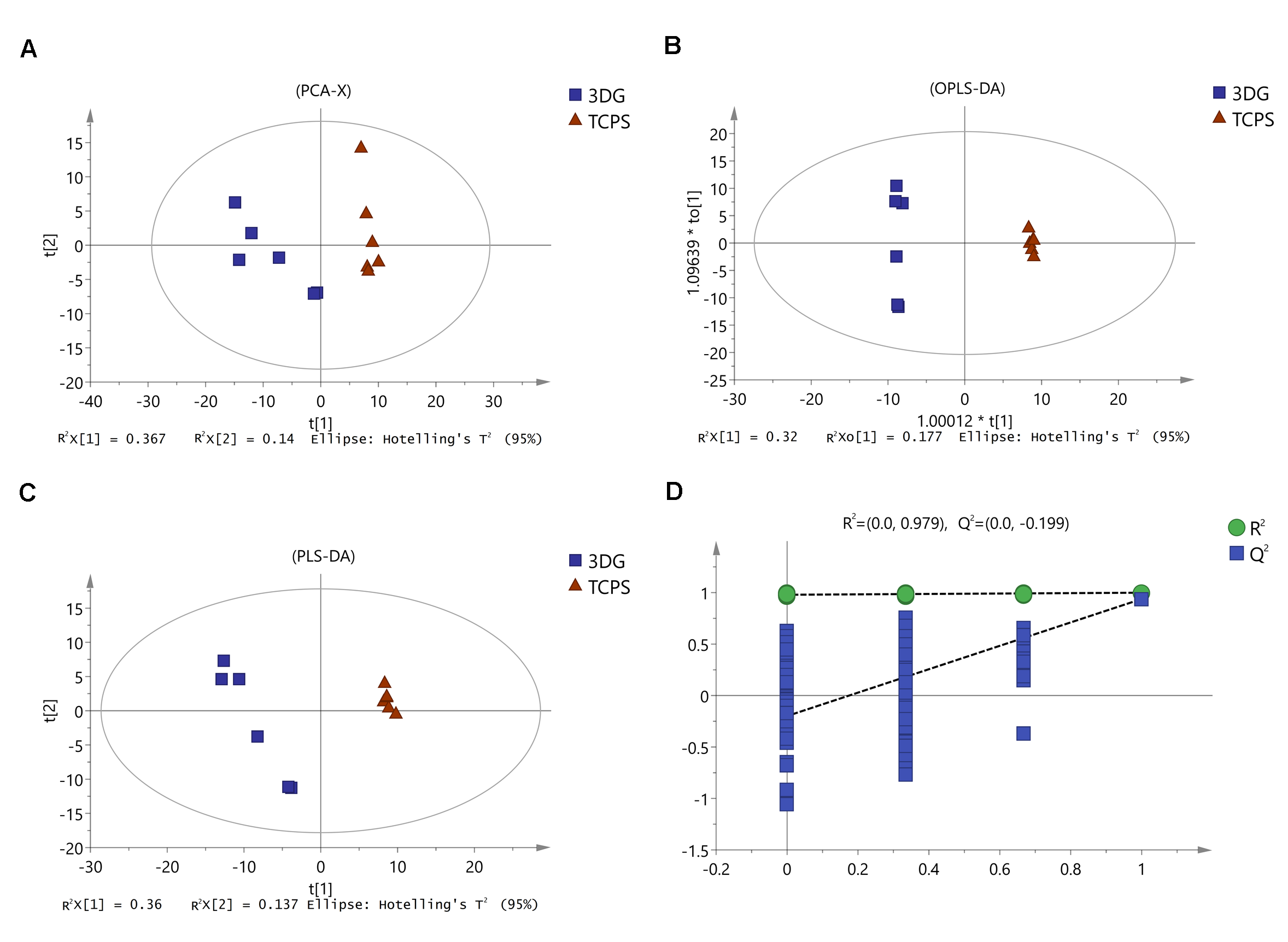

Supplement: Supplementary Figure 2 — Multivariate statistical score graph among three groups. (A) PCA score plot of 3DG and TCPS. (B) OPLS-DA score plot of 3DG and TCPS. (C) PLS-DA score plot of 3DG and TCPS. (D) Statistical validation with permutation analysis (200 times) of the corresponding PLS-DA model of 3DG and TCPS, R2 is the explained variance, and Q2 is the predictive ability of the model. [file Image_2.TIF]

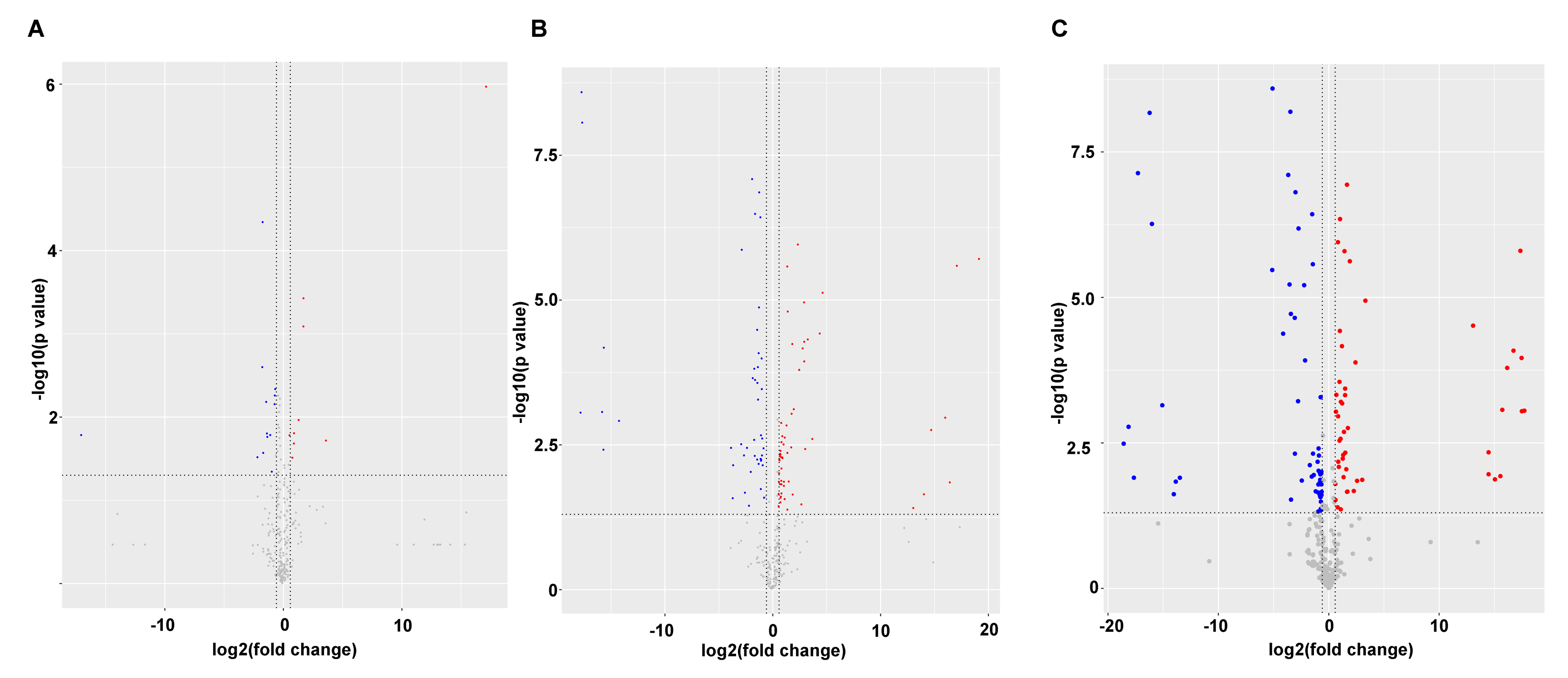

Supplement: Supplementary Figure 3 — volcano map of 3D graphene, 2D graphene and TCPS. (A) 2DG vs. TCPS. (B) 3DG vs. 2DG. (C) 3DG vs. TCPS. up regulation (red), down regulation (green). The horizontal axis is log2 (Fold change), and the vertical axis is –log10 (P-value), each dot represents a gene, and the two lines parallel to the Y axis are X = 1 and X = −1, respectively. The point on the right side of X = 1 is a gene that is up-regulated by a factor of two or more, and the point on the left side of X = −1 is a gene that is down-regulated by a factor of two or more. At the same time, there is a dotted line Y = –log10 (0.05) parallel to the X axis, and a point above the dotted line indicates a gene with a significance P < 0.05. Significantly differentially expressed genes with >2-fold up-regulated genes are marked in red and down-regulated genes are marked in blue. In addition, the difference significance is <2 times and is marked with gray. Moreover, the farther away from the distance between the two axes of X = 1 and X = −1, the greater the variation of the gene represented by this point between the two groups. Through the volcano map, we can clearly see the difference in metabolites of neural stem cells cultured from three different materials. Compared with the 3DG and 2DG groups and the 3DG and TCPS groups, the significant metabolites were significantly better than the 2DG and more than TCPS groups. [file Image_3.TIF]

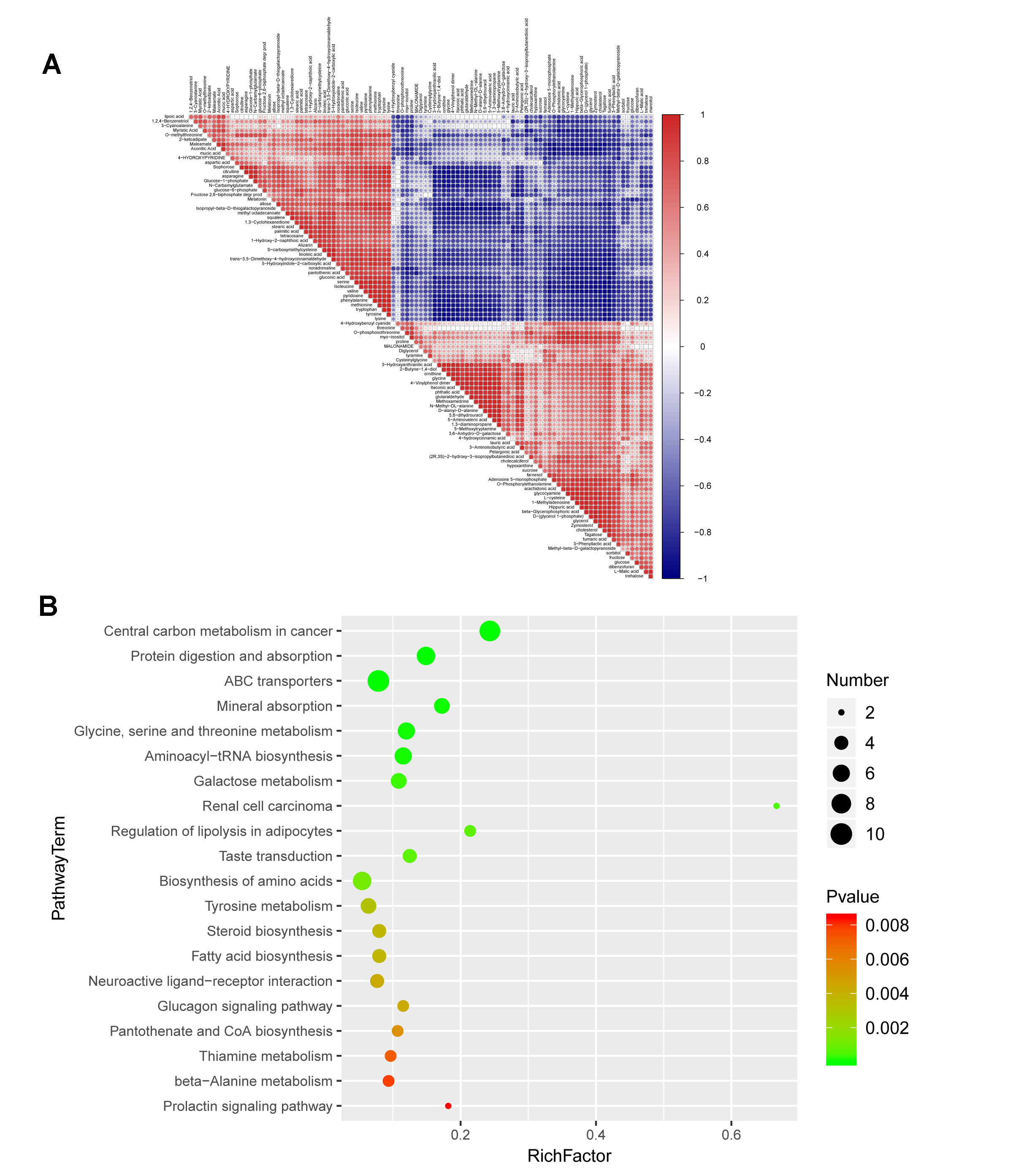

Supplement: Supplementary Figure 4 — (A) Metabolites-metabolites correlations of 3DG vs. TCPS. (B) Bubble diagram of differential metabolic pathways in 3DG vs. TCPS group. [file Image_4.TIF]
