# Supplementary material for: Three-Dimensional Graphene Enhances Neural Stem Cell Proliferation Through Metabolic Regulation
Source: Front Bioeng Biotechnol. 2020 Jan 8;7:436. doi: 10.3389/fbioe.2019.00436 (PMC6961593; doi:10.3389/fbioe.2019.00436)
Supplement: Supplementary Table 1 — Changes in metabolite-related enzymes in two-dimensional graphene and ordinary slides. [file Table_1.docx]

**Supplementary table 1 Changes in metabolite-related enzymes in two-dimensional graphene and ordinary slides**

| ReactionID | Name | Definition | Score |
| --- | --- | --- | --- |
| R02313 | AASS | alpha-aminoadipic semialdehyde synthase | 1.527539438 |
| R02315 | AASS | alpha-aminoadipic semialdehyde synthase | 1.527539438 |
| R00715 | AASS | alpha-aminoadipic semialdehyde synthase | 1.527539438 |
| R00716 | AASS | alpha-aminoadipic semialdehyde synthase | 1.527539438 |
| R00977 | DPYD | dihydropyrimidine dehydrogenase (NADP+) | 1.124254754 |
| R00978 | DPYD | dihydropyrimidine dehydrogenase (NADP+) | 1.124254754 |
| R01795 | phhA, PAH | phenylalanine-4-hydroxylase | 0.854202113 |
| R00031 | TH | tyrosine 3-monooxygenase | 0.854202113 |
| R00731 | TH | tyrosine 3-monooxygenase | 0.854202113 |
| R01815 | TH | tyrosine 3-monooxygenase | 0.854202113 |
| R03539 | TPO | thyroid peroxidase | 0.854202113 |
| R01708 | pdxH, PNPO | pyridoxamine 5'-phosphate oxidase | 0.850717833 |
| R01711 | pdxH, PNPO | pyridoxamine 5'-phosphate oxidase | 0.850717833 |
| R01398 | OTC, argF, argI | ornithine carbamoyltransferase | 0.808108905 |
| R01863 | punA, PNP | purine-nucleoside phosphorylase | 0.786350146 |
| R01128 | hprT, hpt, HPRT1 | hypoxanthine phosphoribosyltransferase | 0.786350146 |
| R01132 | hprT, hpt, HPRT1 | hypoxanthine phosphoribosyltransferase | 0.786350146 |
| R00678 | IDO, INDO | indoleamine 2,3-dioxygenase | 0.76103943 |
| R01814 | TPH1_2 | tryptophan 5-monooxygenase | 0.76103943 |
| R01457 | DHCR24, DWF1 | Delta24-sterol reductase | 0.730524995 |
| R01451 | DHCR7 | 7-dehydrocholesterol reductase | 0.730524995 |
| R01456 | DHCR7 | 7-dehydrocholesterol reductase | 0.730524995 |
| R00762 | FBP, fbp | fructose-1,6-bisphosphatase I | 0.687092159 |
| R07498 | DHCR24, DWF1 | Delta24-sterol reductase | 0.645471315 |
| R07041 | CYP4A | long-chain fatty acid omega-monooxygenase | 0.494956435 |
| R07046 | CYP4A | long-chain fatty acid omega-monooxygenase | 0.494956435 |
| R01595 | ALOX5 | arachidonate 5-lipoxygenase | 0.494956435 |
| R01590 | PTGS1, COX1 | prostaglandin-endoperoxide synthase 1 | 0.494956435 |
| R01593 | ALOX15 | arachidonate 15-lipoxygenase | 0.494956435 |
| R00190 | APRT, apt | adenine phosphoribosyltransferase | 0.311194121 |
| R00127 | AK6, FAP7 | adenylate kinase | 0.311194121 |
| R00181 | AMPD | AMP deaminase | 0.311194121 |
| R01083 | purB, ADSL | adenylosuccinate lyase | -0.311194121 |
| R00183 | E3.1.3.5 | 5'-nucleotidase | -0.311194121 |
| R00185 | E3.1.3.5 | 5'-nucleotidase | -0.311194121 |
| R01596 | ALOX12 | arachidonate 12-lipoxygenase | -0.494956435 |
| R07038 | ALOX12B | arachidonate 12-lipoxygenase (R-type) | -0.494956435 |
| R07053 | ALOX15B | arachidonate 15-lipoxygenase (second type) / 8-lipoxygenase (S-type) | -0.494956435 |
| R07048 | CYP2J | cytochrome P450 family 2 subfamily J | -0.494956435 |
| R07050 | CYP4A | long-chain fatty acid omega-monooxygenase | -0.494956435 |
| R07051 | CYP2J | cytochrome P450 family 2 subfamily J | -0.494956435 |
| R07052 | CYP2J | cytochrome P450 family 2 subfamily J | -0.494956435 |
| R01317 | PLA2G, SPLA2 | secretory phospholipase A2 | -0.494956435 |
| R00851 | GPAT3_4, AGPAT9, AGPAT6 | glycerol-3-phosphate O-acyltransferase 3/4 | -0.639306064 |
| R00841 | glpK, GK | glycerol kinase | -0.639306064 |
| R00847 | glpK, GK | glycerol kinase | -0.639306064 |
| R04804 | EBP | cholestenol Delta-isomerase | -0.645471315 |
| R01068 | ALDO | fructose-bisphosphate aldolase, class I | -0.687092159 |
| R01463 | CYP7A1 | cholesterol 7alpha-monooxygenase | -0.730524995 |
| R01454 | CYP11A | cholesterol monooxygenase (side-chain-cleaving) | -0.730524995 |
| R02723 | CYP11A | cholesterol monooxygenase (side-chain-cleaving) | -0.730524995 |
| R00677 | IL4I1 | L-amino-acid oxidase | -0.76103943 |
| R00684 | IL4I1 | L-amino-acid oxidase | -0.76103943 |
| R10180 | IL4I1 | L-amino-acid oxidase | -0.76103943 |
| R00685 | DDC, TDC | aromatic-L-amino-acid/L-tryptophan decarboxylase | -0.76103943 |
| R01768 | XDH | xanthine dehydrogenase/oxidase | -0.786350146 |
| R01769 | XDH | xanthine dehydrogenase/oxidase | -0.786350146 |
| R02748 | punA, PNP | purine-nucleoside phosphorylase | -0.786350146 |
| R00552 | NOS1 | nitric-oxide synthase, brain | -0.808108905 |
| R00557 | NOS1 | nitric-oxide synthase, brain | -0.808108905 |
| R11711 | NOS1 | nitric-oxide synthase, brain | -0.808108905 |
| R01954 | argG, ASS1 | argininosuccinate synthase | -0.808108905 |
| R01909 | pdxK, pdxY | pyridoxine kinase | -0.850717833 |
| R01911 | pdxK, pdxY | pyridoxine kinase | -0.850717833 |
| R00729 | IL4I1 | L-amino-acid oxidase | -0.854202113 |
| R00734 | IL4I1 | L-amino-acid oxidase | -0.854202113 |
| R09254 | IL4I1 | L-amino-acid oxidase | -0.854202113 |
| R02078 | TYR | tyrosinase | -0.854202113 |
| R02269 | DPYS, dht, hydA | dihydropyrimidinase | -1.124254754 |

**Supplementary table 2 Changes in differential metabolite-related enzymes in three-dimensional graphene and two-dimensional graphene groups**

| ReactionID | Name | Definition | Score |
| --- | --- | --- | --- |
| R01398 | OTC, argF, argI | ornithine carbamoyltransferase | 3.074167521 |
| R00678 | IDO, INDO | indoleamine 2,3-dioxygenase | 3.055253667 |
| R01814 | TPH1_2 | tryptophan 5-monooxygenase | 3.055253667 |
| R00220 | SDS, SDH, CHA1 | L-serine/L-threonine ammonia-lyase | 2.896413697 |
| R00582 | serB, PSPH | phosphoserine phosphatase | 2.896413697 |
| R00585 | AGXT | alanine-glyoxylate transaminase / serine-glyoxylate transaminase / serine-pyruvate transaminase | 2.896413697 |
| R00588 | AGXT | alanine-glyoxylate transaminase / serine-glyoxylate transaminase / serine-pyruvate transaminase | 2.896413697 |
| R00650 | BHMT | betaine-homocysteine S-methyltransferase | 2.536990702 |
| R00946 | BHMT | betaine-homocysteine S-methyltransferase | 2.536990702 |
| R02821 | BHMT | betaine-homocysteine S-methyltransferase | 2.536990702 |
| R04405 | BHMT | betaine-homocysteine S-methyltransferase | 2.536990702 |
| R01795 | phhA, PAH | phenylalanine-4-hydroxylase | 2.30350299 |
| R03539 | TPO | thyroid peroxidase | 2.30350299 |
| R01706 | FASN | fatty acid synthase, animal type | 2.032548606 |
| R01708 | pdxH, PNPO | pyridoxamine 5'-phosphate oxidase | 1.901166986 |
| R01711 | pdxH, PNPO | pyridoxamine 5'-phosphate oxidase | 1.901166986 |
| R00031 | TH | tyrosine 3-monooxygenase | 1.664450626 |
| R00731 | TH | tyrosine 3-monooxygenase | 1.664450626 |
| R01815 | TH | tyrosine 3-monooxygenase | 1.664450626 |
| R08159 | FASN | fatty acid synthase, animal type | 1.629423472 |
| R00977 | DPYD | dihydropyrimidine dehydrogenase (NADP+) | 0.610560636 |
| R00978 | DPYD | dihydropyrimidine dehydrogenase (NADP+) | 0.610560636 |
| R00899 | ANPEP, CD13 | aminopeptidase N | 0.588128993 |
| R02668 | KYNU, kynU | kynureninase | 0.542036091 |
| R02665 | HAAO | 3-hydroxyanthranilate 3,4-dioxygenase | 0.542036091 |
| R03425 | GLDC, gcvP | glycine dehydrogenase | 0.465125271 |
| R00364 | AGXT | alanine-glyoxylate transaminase / serine-glyoxylate transaminase / serine-pyruvate transaminase | 0.465125271 |
| R00366 | AGXT | alanine-glyoxylate transaminase / serine-glyoxylate transaminase / serine-pyruvate transaminase | 0.465125271 |
| R00372 | AGXT | alanine-glyoxylate transaminase / serine-glyoxylate transaminase / serine-pyruvate transaminase | 0.465125271 |
| R06171 | ltaE | threonine aldolase | 0.465125271 |
| R01086 | argH, ASL | argininosuccinate lyase | 0.355499536 |
| R01085 | FAHD1 | acylpyruvate hydrolase | 0.355499536 |
| R02164 | SDHA, SDH1 | succinate dehydrogenase (ubiquinone) flavoprotein subunit | 0.355499536 |
| R01082 | E4.2.1.2B, fumC, FH | fumarate hydratase, class II | 0.274301948 |
| R02038 | PCYT2 | ethanolamine-phosphate cytidylyltransferase | 0.241073435 |
| R01468 | CHK | choline/ethanolamine kinase | 0.241073435 |
| R06516 | SGPL1, DPL1 | sphinganine-1-phosphate aldolase | 0.241073435 |
| R01351 | MGLL | acylglycerol lipase | 0.185467901 |
| R01457 | DHCR24, DWF1 | Delta24-sterol reductase | 0.169891796 |
| R01451 | DHCR7 | 7-dehydrocholesterol reductase | 0.169891796 |
| R01456 | DHCR7 | 7-dehydrocholesterol reductase | 0.169891796 |
| R01001 | CTH | cystathionine gamma-lyase | 0.123003722 |
| R00893 | CDO1 | cysteine dioxygenase | 0.123003722 |
| R00782 | CTH | cystathionine gamma-lyase | 0.123003722 |
| R00895 | GOT1 | aspartate aminotransferase, cytoplasmic | 0.123003722 |
| R00896 | GOT1 | aspartate aminotransferase, cytoplasmic | 0.123003722 |
| R02743 | GGCT | gamma-glutamylcyclotransferase | 0.123003722 |
| R07041 | CYP4A | long-chain fatty acid omega-monooxygenase | 0.115387094 |
| R07046 | CYP4A | long-chain fatty acid omega-monooxygenase | 0.115387094 |
| R01595 | ALOX5 | arachidonate 5-lipoxygenase | 0.115387094 |
| R01590 | PTGS1, COX1 | prostaglandin-endoperoxide synthase 1 | 0.115387094 |
| R01593 | ALOX15 | arachidonate 15-lipoxygenase | 0.115387094 |
| R01187 | E3.1.3.25, IMPA, suhB | myo-inositol-1(or 4)-monophosphatase | 0.097851995 |
| R07279 | E3.1.3.25, IMPA, suhB | myo-inositol-1(or 4)-monophosphatase | 0.097851995 |
| R01186 | E3.1.3.25, IMPA, suhB | myo-inositol-1(or 4)-monophosphatase | 0.097851995 |
| R00841 | glpK, GK | glycerol kinase | 0.062297166 |
| R00847 | glpK, GK | glycerol kinase | 0.062297166 |
| R07498 | DHCR24, DWF1 | Delta24-sterol reductase | 0.04548959 |
| R04804 | EBP | cholestenol Delta-isomerase | -0.04548959 |
| R01802 | CDIPT | CDP-diacylglycerol--inositol 3-phosphatidyltransferase | -0.097851995 |
| R01596 | ALOX12 | arachidonate 12-lipoxygenase | -0.115387094 |
| R07038 | ALOX12B | arachidonate 12-lipoxygenase (R-type) | -0.115387094 |
| R07053 | ALOX15B | arachidonate 15-lipoxygenase (second type) / 8-lipoxygenase (S-type) | -0.115387094 |
| R07048 | CYP2J | cytochrome P450 family 2 subfamily J | -0.115387094 |
| R07050 | CYP4A | long-chain fatty acid omega-monooxygenase | -0.115387094 |
| R07051 | CYP2J | cytochrome P450 family 2 subfamily J | -0.115387094 |
| R07052 | CYP2J | cytochrome P450 family 2 subfamily J | -0.115387094 |
| R01317 | PLA2G, SPLA2 | secretory phospholipase A2 | -0.115387094 |
| R00894 | GCLC | glutamate--cysteine ligase catalytic subunit | -0.123003722 |
| R07460 | iscS, NFS1 | cysteine desulfurase | -0.123003722 |
| R00851 | GPAT3_4, AGPAT9, AGPAT6 | glycerol-3-phosphate O-acyltransferase 3/4 | -0.123170735 |
| R01463 | CYP7A1 | cholesterol 7alpha-monooxygenase | -0.169891796 |
| R01454 | CYP11A | cholesterol monooxygenase (side-chain-cleaving) | -0.169891796 |
| R02723 | CYP11A | cholesterol monooxygenase (side-chain-cleaving) | -0.169891796 |
| R00748 | AGXT2L1, ETNPPL | ethanolamine-phosphate phospho-lyase | -0.241073435 |
| R01364 | FAH, fahA | fumarylacetoacetase | -0.355499536 |
| R00751 | ltaE | threonine aldolase | -0.465125271 |
| R00497 | GSS | glutathione synthase | -0.465125271 |
| R00830 | E2.3.1.37, ALAS | 5-aminolevulinate synthase | -0.465125271 |
| R00610 | PIPOX | sarcosine oxidase / L-pipecolate oxidase | -0.465125271 |
| R00611 | PIPOX | sarcosine oxidase / L-pipecolate oxidase | -0.465125271 |
| R01221 | GLDC, gcvP | glycine dehydrogenase | -0.465125271 |
| R03630 | ADAM29 | disintegrin and metalloproteinase domain-containing protein 29 | -0.53259549 |
| R02269 | DPYS, dht, hydA | dihydropyrimidinase | -0.610560636 |
| R00214 | E1.1.1.40, maeB | malate dehydrogenase (oxaloacetate-decarboxylating)(NADP+) | -0.629801483 |
| R00216 | E1.1.1.40, maeB | malate dehydrogenase (oxaloacetate-decarboxylating)(NADP+) | -0.629801483 |
| R00342 | MDH2 | malate dehydrogenase | -0.629801483 |
| R00342 | MDH2 | malate dehydrogenase | -0.629801483 |
| R00361 | MDH2 | malate dehydrogenase | -0.629801483 |
| R02080 | DDC, TDC | aromatic-L-amino-acid/L-tryptophan decarboxylase | -0.639052365 |
| R01909 | pdxK, pdxY | pyridoxine kinase | -1.901166986 |
| R01911 | pdxK, pdxY | pyridoxine kinase | -1.901166986 |
| R01274 | ACSL, fadD | long-chain acyl-CoA synthetase | -2.032548606 |
| R01280 | ACSL, fadD | long-chain acyl-CoA synthetase | -2.032548606 |
| R00729 | IL4I1 | L-amino-acid oxidase | -2.30350299 |
| R00734 | IL4I1 | L-amino-acid oxidase | -2.30350299 |
| R09254 | IL4I1 | L-amino-acid oxidase | -2.30350299 |
| R07324 | INO1, ISYNA1 | myo-inositol-1-phosphate synthase | -2.397178129 |
| R00945 | glyA, SHMT | glycine hydroxymethyltransferase | -2.431288426 |
| R00648 | IL4I1 | L-amino-acid oxidase | -2.536990702 |
| R07396 | IL4I1 | L-amino-acid oxidase | -2.536990702 |
| R00177 | metK | S-adenosylmethionine synthetase | -2.536990702 |
| R01281 | SPT | serine palmitoyltransferase | -2.896413697 |
| R01290 | CBS | cystathionine beta-synthase | -2.896413697 |
| R00589 | SRR | serine racemase | -2.896413697 |
| R02078 | TYR | tyrosinase | -2.942555355 |
| R00677 | IL4I1 | L-amino-acid oxidase | -3.055253667 |
| R00684 | IL4I1 | L-amino-acid oxidase | -3.055253667 |
| R10180 | IL4I1 | L-amino-acid oxidase | -3.055253667 |
| R00685 | DDC, TDC | aromatic-L-amino-acid/L-tryptophan decarboxylase | -3.055253667 |
| R00552 | NOS1 | nitric-oxide synthase, brain | -3.074167521 |
|  | NOS1 | nitric-oxide synthase, brain | -3.074167521 |
| R11711 | NOS1 | nitric-oxide synthase, brain | -3.074167521 |
| R01954 | argG, ASS1 | argininosuccinate synthase | -3.074167521 |

**Supplementary table 3 Metabolic pathways and the KEGG ID in this study**

| mmu00400 | Phenylalanine, tyrosine and tryptophan biosynthesis |
| --- | --- |
| mmu05230 | Central carbon metabolism in cancer |
| mmu01230 | Biosynthesis of amino acids |
| mmu04925 | Aldosterone synthesis and secretion |
| mmu04913 | Ovarian steroidogenesis |
| mmu05217 | Basal cell carcinoma |
| mmu04726 | Serotonergic synapse |
| mmu04974 | Protein digestion and absorption |
| mmu00260 | Glycine, serine and threonine metabolism |
| mmu00100 | Steroid biosynthesis |
| mmu00970 | Aminoacyl-tRNA biosynthesis |
| mmu04916 | Melanogenesis |
| mmu04912 | GnRH signaling pathway |
| mmu05140 | Leishmaniasis |
| mmu05030 | Cocaine addiction |
| mmu05143 | African trypanosomiasis |
| mmu04666 | Fc gamma R-mediated phagocytosis |
| mmu02010 | ABC transporters |
| mmu04917 | Prolactin signaling pathway |
| mmu05211 | Renal cell carcinoma |
| mmu04923 | Regulation of lipolysis in adipocytes |
| mmu05012 | Parkinson disease |
| mmu00350 | Tyrosine metabolism |
| mmu04922 | Glucagon signaling pathway |
| mmu05031 | Amphetamine addiction |
| mmu00730 | Thiamine metabolism |
| mmu04742 | Taste transduction |
| mmu05034 | Alcoholism |
| mmu05200 | Pathways in cancer |
| mmu01200 | Carbon metabolism |
| mmu04978 | Mineral absorption |
| mmu00052 | Galactose metabolism |
| mmu00061 | Fatty acid biosynthesis |
| mmu04080 | Neuroactive ligand-receptor interaction |
| mmu00770 | Pantothenate and CoA biosynthesis |
| mmu00410 | beta-Alanine metabolism |
